# Milk Thistle Extract and Silymarin Inhibit Lipopolysaccharide Induced Lamellar Separation of Hoof Explants *in Vitro*

**DOI:** 10.3390/toxins6102962

**Published:** 2014-10-06

**Authors:** Nicole Reisinger, Simone Schaumberger, Veronika Nagl, Sabine Hessenberger, Gerd Schatzmayr

**Affiliations:** 1 Biomin Research Center, Technopark 1, Tulln 3430, Austria; E-Mails: simone.schaumberger@biomin.net (S.S.); sabine.hessenberger@biomin.net (S.H.); gerd.schatzmayr@biomin.net (G.S.); 2 Center for Analytical Chemistry, Department for Agrobiotechnology (IFA Tulln), University of Natural Resources and Life Sciences, Vienna, Tulln (BOKU), Konrad Lorenz Str. 20, Tulln 3430, Austria; E-Mail: veronika.nagl@boku.ac.at

**Keywords:** horses, equine, endotoxins, laminitis, hoof explants, milk thistle, silymarin

## Abstract

The pathogenesis of laminitis is not completely identified and the role of endotoxins (lipopolysaccharides, LPS) in this process remains unclear. Phytogenic substances, like milk thistle (MT) and silymarin, are known for their anti-inflammatory and antioxidant properties and might therefore have the potential to counteract endotoxin induced effects on the hoof lamellar tissue. The aim of our study was to investigate the influence of endotoxins on lamellar tissue integrity and to test if MT and silymarin are capable of inhibiting LPS-induced effects in an *in vitro*/*ex vivo* model. In preliminary tests, LPS neutralization efficiency of these phytogenics was determined in an *in vitro* neutralization assay. Furthermore, tissue explants gained from hooves of slaughter horses were tested for lamellar separation after incubation with different concentrations of LPS. By combined incubation of explants with LPS and either Polymyxin B (PMB; positive control), MT or silymarin, the influence of these substances on LPS-induced effects was assessed. In the *in vitro* neutralization assay, MT and silymarin reduced LPS concentrations by 64% and 75%, respectively, in comparison PMB reduced 98% of the LPS concentration. In hoof explants, LPS led to a concentration dependent separation. Accordantly, separation force was significantly decreased by 10 µg/mL LPS. PMB, MT and silymarin could significantly improve tissue integrity of explants incubated with 10 µg/mL LPS. This study showed that LPS had a negative influence on the structure of hoof explants *in vitro*. MT and silymarin reduced endotoxin activity and inhibited LPS-induced effects on the lamellar tissue. Hence, MT and silymarin might be used to support the prevention of laminitis and should be further evaluated for this application.

## 1. Introduction

Laminitis is one of the most common diseases in horses and has significant influence on the horse industry [1]. The pathogenesis of equine laminitis has a multifactorial etiology and is yet not fully understood. Bacterial exotoxins [2,3] as well as bacterial endotoxins [4,5] have been suggested to contribute to the pathophysiology of laminitis. Endotoxins, also known as lipopolysaccharides (LPS), are a major component of the outer leaflet of the outer membrane of Gram-negative bacteria and are released during death or excessive growth of bacteria.

Several studies investigated the role of endotoxins in the pathogenesis of laminitis. For example, significant plasma LPS concentrations could be detected after experimental induced laminitis using a carbohydrate overload model [4,6]. Perfusion of forelimbs with LPS for 10 h showed morphological changes of the lamellar tissue and metabolic imbalances [7]. However, administration of endotoxins alone failed to induce laminitis [8,9]. Drawbacks of these *in vivo* and perfusion studies are the single or short time administrations of LPS, which do not reflect the continuous exposure to LPS during intestinal disturbance. In contrast, hoof explant models, as established by Pollitt *et al.* or Pass *et al.* [10,11] provide a novel tool to investigate the pathogenesis of laminitis. Pass *et al.* [10] noted that the separation of the basal epidermal cells from their basement membrane in this model mimics the pathology of laminitis and can therefore be used to test potential trigger factors. For example, influence of bacterial exotoxins has already been tested in the hoof explant model [2,3]. Comparable data for endotoxins in this model are lacking so far, but would provide substantial information concerning the pathogenesis of laminitis.

After identification of contribution factors of laminitis, it is important to find substances which can inhibit the negative influence of toxins on lamellar tissue. Polymyxin B (PMB) is an antibiotic capable of deactivating circulating endotoxins. However, studies about its effects on experimentally induced laminitis are rare. Due to its nephrotoxic and neurotoxic effects, its therapeutic use is limited [12]. Hence, there is an urgent need to find alternative, non-antibiotic substances which are effective against LPS, especially for preventive use. As laminitis is an inflammation of the lamellar tissue, phytogenic substances with anti-inflammatory properties might have the potential to neutralize LPS in the gut. Milk thistle (*Silybum marianum*) is known to have positive influence on the detoxification activity of the liver [13,14]. Silymarin, a standardized extract of milk thistle, and silybinin, the major active component of silymarin, both showed anti-inflammatory [15] and antioxidant properties [16] *in vivo* and *in vitro.* There is no scientific literature available elucidating whether milk thistle extracts are able to directly neutralize LPS or if they exhibit positive effects on the lamellar tissue.

The aim of our study was to evaluate the effects of milk thistle and silymarin in an established *ex vivo*/*in vitro* laminitis model, using LPS as trigger factor and PMB as control. Moreover, it was tested if milk thistle extract and silymarin can neutralize LPS in a similar way like PMB in an *in vitro* neutralization assay.

## 2. Results

### 2.1. In Vitro Endotoxin Neutralization Tests

**Figure 1:**
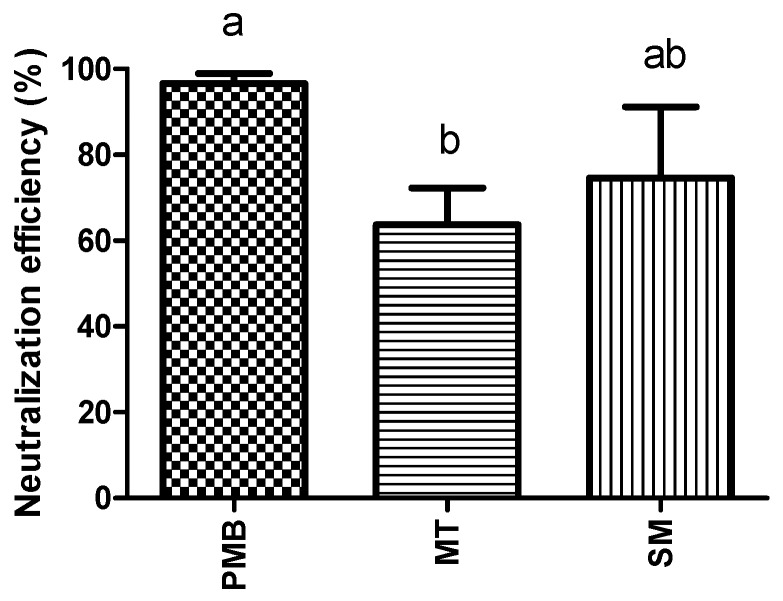
Neutralization efficiency (%) of polymyxin (PMB), milk thistle (MT) and silymarin (SM) was tested with a LPS solution with a measured endotoxin activity of 43,533 EU/mL (±2433 EU/mL) at 37 °C for 2 h (*n* = 3 independent experiments). Error bars display standard deviation. ^a,b^ Superscripts indicate significant difference *p* < 0.05.

An *in vitro* endotoxin neutralization test was used to test the neutralization efficiency of PMB, milk thistle (MT) and silymarin. Endotoxin activity is expressed in endotoxin units (EU). One ng LPS is equivalent to 7 EU. In three independent experiments, substances were incubated with an LPS solution with a measured endotoxin activity of 43,533 EU/mL (±2433 EU/mL). All substances significantly reduced the LPS concentration (*p* < 0.005). PMB had a significantly increased neutralization efficiency (97%) compared to MT (64%), whereas differences to silymarin (75%) were not statistically significant (Figure 1).

### 2.2. Viability

**Figure 2:**
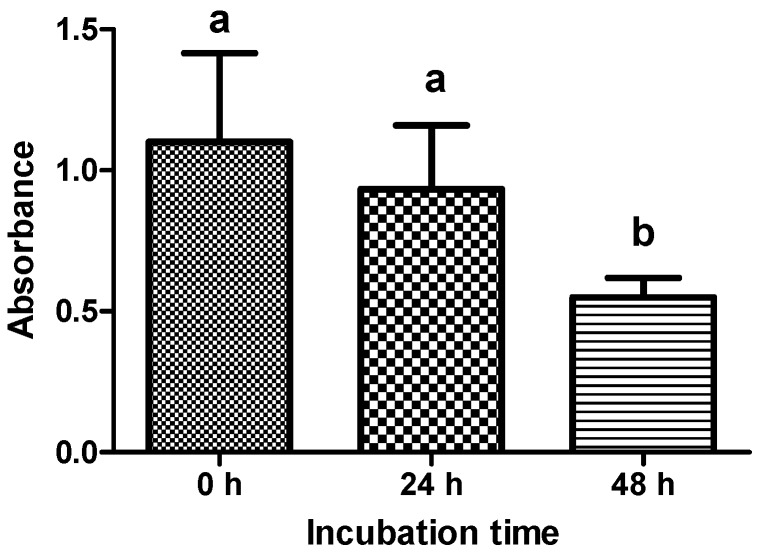
Absorbance values at 450 nm measured with the WST-1 assay (*n* = 12 explants per treatment) to evaluate viability of explants after dissection, after 24 h and 48 h incubation with medium. Error bars display standard deviation. ^a,b^ Superscripts indicate significant difference *p* < 0.05.

A viability test was performed to ensure that explants dissected from the hooves were still viable after cultivation. Supernatants of explants were tested with the water soluble tetrazolium (WST-1) assay after dissection, 24 and 48 h of incubation (*n* = 12 explants per treatment). Supernatants of all control explants showed an increase of absorbance and were therefore considered as viable. Explants incubated for 48 h showed a significant decrease in viability compared to explants tested immediately after dissection or 24 h of incubation (*p* < 0.005) (Figure 2).

**Table 1:** Number of separated explants after LPS treatment (1.25–200 µg/mL) for 24 and 48 h. Contaminated explants (eight out of 293 explants in total) were excluded from results.

	Number of explants	
	Separated/total	Separated/total
LPS (µg/mL)	24 h	48 h
0	3/18 ^a^	0/32 ^a^
1.25	n.d.	0/8 ^a^
2.5	5/10 ^a^	0/12 ^a^
5	16/18 ^b^	4/12 ^a^
10	18/18 ^b^	6/12 ^a^
20	16/17 ^b^	11/11 ^b^
40	18/18 ^b^	12/12 ^b^
60	n.d.	8/8 ^b^
80	n.d.	9/9 ^b^
100	18/18 ^b^	12/12 ^b^
120	n.d.	9/9 ^b^
140	n.d.	9/9 ^b^
160	n.d.	9/9 ^b^
180	n.d.	9/9 ^b^
200	18/18 ^b^	12/12 ^b^

^a,b^ Superscripts indicate significant difference *p* < 0.05.

### 2.3. Studies with LPS

Hoof explants were dissected from different horses to measure tissue integrity after *in vitro* cultivation with LPS. To this end, hoof explants were incubated in triplicate with different concentrations of LPS for 24 and 48 h. Structural integrity was evaluated with two rat tooth forceps. Number of separated explants (six hooves) incubated with medium (negative control) for 24 h was significantly lower compared to explants incubated with 5–200 µg/mL LPS (Table 1). Furthermore, number of separated explants (four hooves) incubated with medium for 48 h was significantly lower compared to explants incubated with 20–200 µg/mL LPS (Table 1).

### 2.4. Studies with LPS in Combination with PMB

Explants of 10 hooves were used to test the capability of PMB to inhibit the lamellar separation induced by LPS. Structural integrity was evaluated with two rat tooth forceps. Compared to the positive control (incubation with 10 µg/mL LPS), addition of 50–500 µg/mL PMB significantly decreased the number of separated explants (Table 2). No concentration dependent effect of PMB could be observed. If applied alone, PMB concentrations (50–500) used did not lead to lamellar separation (data not shown).

**Table 2:** Number of separated explants after treatment with medium, LPS (10 µg/mL) and LPS (10 µg/mL) combined with PMB (50–500 µg/mL) for 24 h. Contaminated explants (three out of 126 explants in total) were excluded from results.

Treatment		Number of explants
LPS (µg/mL)	PMB (µg/mL)	Separated/total
0	0	3/30 ^a^
10	0	28/30 ^b^
10	50	2/8 ^a^
10	100	0/9 ^a^
10	200	6/20 ^a^
10	500	3/26 ^a^

^a,b^ Superscripts indicate significant difference *p* < 0.05.

### 2.5. Studies with LPS in Combination with MT and Silymarin

#### 2.5.1. Results of Forceps Testing

As a first step, MT and silymarin were tested in different concentrations for their potential to reduce LPS induced lamellar separation (four hooves). Structural integrity was evaluated with two rat tooth forceps. Incubation of explants with 500 and 1000 µg/mL MT significantly decreased the number of separated explants (Table 3). Also, incubation of explants with 100 and 250 µg/mL silymarin significantly decreased the number of separated explants compared to explants incubated only with 10 µg/mL LPS (Table 3). If applied alone, tested concentrations of MT (10–1000 µg/mL) and silymarin (100 and 250 µg/mL) did not lead to lamellar separation (data not shown).

#### 2.5.2. Force Transducer Results

**Figure 3:**
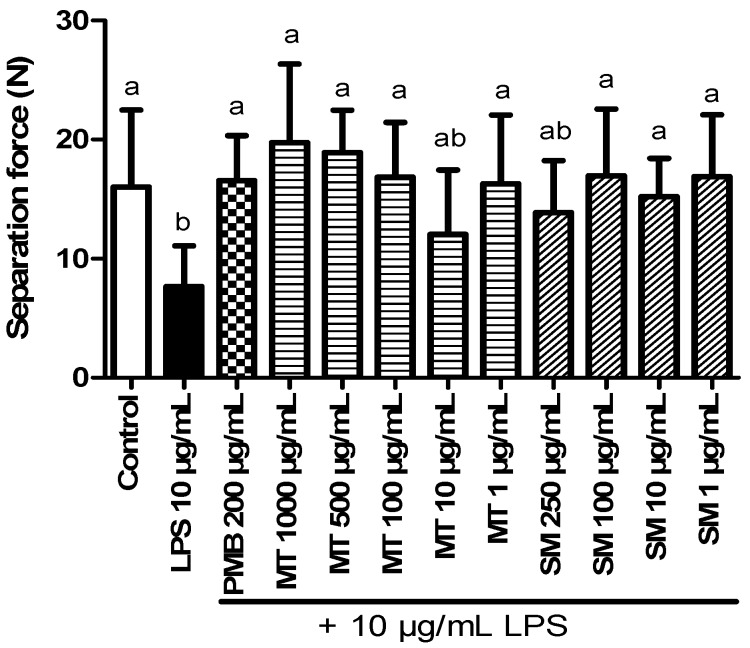
Explants incubated with medium (control), 10 µg/mL LPS, polymyxin B (PMB) (200 µg/mL), milk thistle (MT) (1–1000 µg/mL) and silymarin (SM) (1–250 µg/mL) combined with 10 µg/mL LPS. Error bars display standard deviation. ^a,b^ Superscripts indicate significant difference *p* < 0.05.

In addition, effects of LPS on tissue integrity were tested with a force transducer. Separation force was significantly decreased in explants incubated with 10 µg/mL LPS (*n* = 24) (7.7 ± 3.4 Newton (N)) compared to explants (*n* = 29) incubated with medium alone (16.0 ± 6.5 N) (Figure 3). Furthermore, influence of PMB, MT and silymarin on separation force of explants treated with 10 µg/mL LPS was evaluated (two hooves). PMB (200 µg/mL) inhibited the decrease of separation force caused by LPS (Figure 3). Silymarin generally increased the separation force compared to LPS. Statistically significant differences were observed at levels of 1 and 100 µg/mL silymarin, but no concentration dependent effect could be determined. In contrast, MT (1, 100, 500, 1000 µg/mL) increased the separation force in a concentration dependent matter.

**Table 3:** Number of separated explants after treatment with medium, LPS (10 µg/mL), MT (10–1000 µg/mL) combined with LPS (10 µg/mL) and silymarin (100 and 250 µg/mL) combined with LPS (10 µg/mL) for 24 h. Contaminated explants (three out of 66 explants in total) were excluded from results.

Treatment		Number of explants
LPS (µg/mL)	MT, silymarin (µg/mL)	Separated/total
0	0	0/8 ^a^
10	0	9/9 ^b^
10	MT 10	4/6 ^b^
10	MT 100	3/9 ^b^
10	MT 500	0/9 ^a^
10	MT 1000	2/9 ^a^
10	Silymarin 100	0/3 ^a^
10	Silymarin 250	0/5 ^a^

^a,b^ Superscripts indicate significant difference *p* < 0.05.

## 3. Discussion

As laminitis has multifactorial etiology, various events (e.g., oligosaccharide or starch overload) and different trigger factors (e.g., bacterial toxins) are associated with this disease. Our study focused on the involvement of bacterial endotoxins during the pathogenesis of laminitis using an *ex vivo*/*in vitro* model.

Although increased plasma endotoxin levels were measured in experimentally induced laminitis [4,6] and pretreatment of horses with LPS increased the incidence and severity of this disease [17], intravenous administration of LPS alone failed to induce laminitis [9,18]. Nevertheless, administration of endotoxins demonstrated negative effects on hooves and other clinical parameters *in vivo*, e.g., increased heart rate [19,20]. These studies strengthen the hypothesis that LPS is not able to induce laminitis on its own, but plays an important role during its pathogenesis. There is still no *in vivo* model available, which mimics the complex process of naturally occurring laminitis. In particular, the continuous exposure of the hoof to LPS remains a challenge under these conditions. The explant model used in our study was used as a tool to investigate effects of endotoxins with an increased exposure time. Furthermore, it provides a good opportunity to test single trigger factors and mimic lamellar separation *in vitro*.

Our study demonstrated that endotoxins have a negative effect on the lamellar tissue. Compared to control explants, incubation with LPS concentrations of 5–100 µg/mL significantly increased the number of separated explants as well as decreased separation force. Explants showed the typical dermal-epidermal separation, which is characteristic for lamellar separation in course of laminitis. The LPS concentrations used in our study are comparable to those administered in *in vivo* studies. For example, Duncan *et al.* [19] administered 12 mg LPS per horse for 24 h to induce hoof discomfort, while Ingle-Fehr and Baxter [20] used 50 µg per horse to cause changes in the digital blood flow. However, it is difficult to compare our *in vitro* study to *in vivo* studies, as LPS concentrations administered are related to body weight. Other *in vitro* studies used comparable concentrations of LPS to stimulate equine cells [21,22]. Unfortunately, reports on tissue cultivation with LPS are very rare. Mungall *et al.* [2] tested supernatants of Gram-positive and Gram-negative bacteria cultures in the explant model. All supernatants led to separation of explants. Addition of LPS alone also led to lamellar separation in this study, but in contrast to our experiments, no concentration dependent separation could be observed. Since no data on used concentrations or separation force were published, it is difficult to compare our results with this study. Thus, our study provides the first results on direct effects of defined LPS concentrations on the lamellar tissue of cultivated hoof explants and gives further evidence for the importance of LPS in the pathogenesis of laminitis.

To investigate possible time-dependent effects of LPS exposure, separation tests were performed after 24 and 48 h of incubation. Interestingly, at certain concentrations, influence of LPS was more prominent after 24 h. There are different hypotheses for this observation: (1) Viability tests revealed a significantly decreased viability of explants after 48 h. Thus, the induction of certain intracellular cascades e.g., cytokine production, could be impaired by prolonged incubation times, making the cells less sensitive to LPS treatment; (2) Cells started to repair damage induced by LPS and dealt with its effects. Our data support the second theory because described effects were only seen at low concentrations (2.5–10 µg/mL). Our results emphasize the importance of monitoring explant viability in this *ex vivo*/*in vitro* model.

Since our results showed that endotoxins have a negative influence on integrity of hoof explants, we tested certain substances for their potential to counteract LPS induced effects. PMB was included in our experiments, as for a long time, PMB has been known to bind endotoxins. In accordance, PMB neutralized nearly 100% of LPS in our *in vitro* neutralization assay. It also inhibited LPS induced effects, as less explants separated and separation force increased in samples treated with PMB. There are only a few reports available focusing on the effects of PMB application during endotoxaemia in horses [23,24]. In these studies, PMB seems to provide a sufficient treatment of healthy horses challenged with LPS. However, cases of severe side effects of PMB treatment are described in literature, e.g., transient tachypnea, increased plasma thromboxane B2 [18] and ataxic gait [25]. Hence, alternative strategies for treatment or even prevention of endotoxin related diseases are necessary.

We therefore investigated the effects of phytogenic substances on LPS induced lamellar separation. We tested MT and silymarin in our study due to their potential to inhibit the absorption of toxins [13,26] and their low toxicity [26,27]. Silymarin and silibin, the major active part of silymarin, show hepatoprotective [14,28] antifibrotic [29,30], anti-inflammatory [15,26] and antioxidant effects [16,31] in humans and animals. Because of these characteristics, MT and its extracts might indirectly facilitate detoxification of endotoxins or reduction of LPS induced effects. Especially, anti-inflammatory and antioxidant effects of MT might be important, as laminitis is associated with inflammation of the lamellar tissue. However, the ability of these substances to directly neutralize LPS has never been investigated. In our *in vitro* neutralization assay, MT showed a reduction of the endotoxin activity, but the decrease was significantly lower compared to PMB. Notably, neutralization efficiency of silymarin was comparable to that of PMB. In the explant model, MT and silymarin were both able to inhibit LPS (10 µg/mL) induced lamellar separation. Silymarin is a standardized extract of MT. Therefore, lower concentrations were necessary to be as or even more effective than the self-prepared extract of MT. A possible explanation for the neutralization of LPS by MT and silymarin is based on their structure, which might lead to binding and/or structural changes of LPS with subsequent inactivation of the toxin. Beside direct neutralization, we conclude that MT and silymarin were also effective against indirect effects (e.g., oxidative stress, impaired glucose metabolism) of LPS. There is no scientific literature available on the direct neutralization of endotoxins by MT or silymarin or the influence of these substances during laminitis. Nevertheless, milk thistle has already been used in many feed additives in the equine industry, and also in additives claiming to be effective against laminitis. Our study therefore presents the first steps to prove that MT can inhibit negative influence of LPS on the lamellar tissue. A crucial point that should be considered when feeding MT is the actual content of silymarin and silibinin in the administered product. To determine suitable concentrations, as well as to answer important questions regarding mode of action and effects against other laminitis trigger factors, further *in vitro* and *in vivo* studies are required.

## 4. Experimental Section

### 4.1. In Vitro LPS Neutralization Assay and Limulus Amebocyte Lysate (LAL) Assay

For the *in vitro* LPS neutralization assay, 5 mg of polymyxin B (Bioreagent, Sigma Aldrich,Vienna, Austria), milk thistle and silymarin (Sigma Aldrich, Vienna, Austria) were weighed in depyrogenized borosilicate glass tubes (Pyrokontrol^®^, ACILA, Weiterstadt, Germany).

Afterwards, 5 mL of LAL-reagent water (Lonza, Basel, Switzerland) and LPS from *Escherichia coli* O55:B5 (Sigma Aldrich) (calculated 10,000 EU/mL) were added. Solutions were incubated for 2 h at 37 °C at 112 × *g*. After incubation, solutions were heat inactivated for 15 min at 100 °C in a water bath and centrifuged at 500 × *g* for 15 min thereafter. Endotoxin concentrations (EU/mL) of the supernatants were measured with the LAL assay (Charles River, Charleston, WV, USA). All materials used for the LAL assay were pyrogen-free. Glucashield buffer (Cape God, Pyroquant, Mörfelden-Walldorf, Germany) was used to block the interference of (1→3)-β-d-glucans in the LAL assay. The endotoxin activities were calculated using Endo-ScanV software (9.1, Charles River, Charleston, WV, USA, 2012). Neutralization efficiency was calculated in percentage (%), where *N* was the neutralization efficiency and *C*_0_ and *C* were the endotoxin concentrations measured in the stock solution and in sample supernatant, respectively.

(1) $$N=\left(\frac{C_{0}-C}{C_{0}}\right)\times 100$$

### 4.2. Animals

Forelimbs (*n* = 24) from 18 adult horses were obtained at local abattoir. Information on age, gender, breed or history was not available. Horses were killed by use of a penetrating captive bolt. After exsanguination, the forelimbs were disarticulated at the middle carpal joint. Only hooves with no sign of hoof diseases were used. Evaluation of hoof condition and macroscopic evaluation of lamella were performed by a veterinarian. The forelimbs were transported on ice to the laboratory. Time from death of the horses until arrival of the isolated forelimbs at the laboratory did not exceed 120 min.

### 4.3. Preparation of Explants

Prior to dissection, hooves were cleaned with an antibacterial disinfectant (Unigloves, Siegburg, Germany). Dissection of the hooves was performed as described by Pollitt [32]. Hoof explants consisted of 2 mm of the inner hoof wall, 6–8 intact epidermal lamella junctions and 2 mm of dermal connective tissue. Before cultivation, the explants were washed three times with sterile sodium chloride solution (0.9%) (Sigma Aldrich, Vienna, Austria) and once in sterile phosphate buffered saline solution (PBS) (Gibco, Life technologies, Vienna, Austria) under sterile conditions.

### 4.4. Preparation of Endotoxin, PMB, MT and Silymarin Stock Solutions

Stock solutions of LPS and PMB, containing 1 mg/mL, were prepared by dissolving LPS from *Escherichia coli* O55:B5 and PMB, respectively, in sterile PBS. Milk thistle press cake (local plant supplier, Lower Austria, Austria) was ground and extracted with the 10-fold amount of ethanol/water (70/30; *v*/*v*) for 48 h on a rotary shaker (IKA, Staufen, Germany). Afterwards, the liquid extract was filtrated and ethanol was subsequently removed by the use of a rotary evaporator (Heidolph, Schwabach, Germany). After lyophylization, the extract was dissolved in ethanol/water (70/30, *v*/*v*) to yield a concentration of 50 mg/mL. Silymarin was dissolved in dimethyl sulfoxide (DMSO) (Sigma Aldrich, Vienna, Austria) to a final concentration of 10 mg/mL.

### 4.5. Cultivation

Explants were cultured in triplicates in 24 well plates (IWAKI, Willich, Germany) with 1 mL medium at 37 °C and 5% CO_2_ for 24 or 48 h. D-MEM (Gibco, Life technologies, Vienna, Austria), supplemented with 4.5 g/L glucose, 0.1 mg/mL gentamicin (Gibco, Life technologies, Vienna, Austria) and 100 U/mL nystatin (Gibco, Life technologies, Vienna, Austria), was used as medium. To investigate the influence of LPS, PMB, MT and silymarin on the structural integrity of hoof explants, the following experiments were performed: In the first study, different concentrations of LPS (1.25–200 µg/mL) were added to the explants for 24 (six hooves) and 48 h (four hooves). In the second experiment, the explants were cultured with PMB (50–500 µg/mL) alone as well as with PMB combined with 10 µg/mL LPS for 24 h (*n* = 10 hooves). In a third experiment, the effects of MT (1–1000 µg/mL) or silymarin (100 and 250 µg/mL) alone or in combination with LPS (10 µg/mL) were tested (four hooves) for 24 h.

Explants cultured in medium were used as negative control in all trials. In addition, it was verified that all solvents used (PBS, ethanol (70/30, *v*/*v*) and DMSO) had no effect on the explants. Bacterial or fungal contamination of explants was determined via microscopic evaluation. Contaminated explants were excluded from the trial.

### 4.6. Viability

Control explants (*n* = 3) of every hoof were tested for viability with the WST-1 assay (Roche, Vienna, Austria). WST-1 reagent diluted with medium (10/90; *v*/*v*) was therefore added to each well, and incubated for 2 h at 37 °C. Explants were considered as viable, if color of explant supernatants changed from light red to orange. If control explants were not viable, the respective hoof was excluded from the trial. Furthermore, the absorbance of the supernatants was measured at 450 nm to compare the viability after different incubation times. Explants were tested immediately after dissection as well as after 24 and 48 h of incubation.

### 4.7. Separation

Explants were tested for their structural integrity with two rat tooth forceps after 24 and 48 h as described by Pollitt *et al.* [11]. All explants were tested by the same operator.

In addition, tissue integrity was evaluated by measuring the force that is necessary to separate the explants (= separation force) treated with LPS, PMB, MT and silymarin. The explants were therefore immobilized on one side, while the other side was connected to a calibrated force transducer (Sautner, BatschWaagen und EDV, Loosdorf, Austria) with a mosquito clamp. The maximum load to failure was measured in Newton.

### 4.8. Statistical Analyses

Statistical evaluation was performed using commercial available IBM SPSS statistics software (Version 19.0, IBM corp., New York, US, 2010). ANOVA was used for results of the *in vitro* neutralization assay with Bonferroni’s Multiple Comparison Test as *post-hoc* test. For pair-wise comparisons of results from manual explant separation test, separation force and viability, the Kruskal Wallis Test was used as non-parametric test. Results were considered significant at *p* < 0.05.

## 5. Conclusions

We confirmed that LPS has a negative influence on the structure of the hoof explants *in vitro*, and therefore should be further screened for its contribution during the pathogenesis of laminitis. MT and silymarin were not only able to neutralize endotoxins, but also capable of reducing LPS-induced lamellar separation. Hence, MT and silymarin might be used to support the prevention of laminitis through, direct neutralization of endotoxins and inhibition of LPS induced effects on the lamellar tissue. However, further investigations on endotoxins and their contribution during development of laminitis are necessary. In addition, the mode of action of MT and silymarin on LPS neutralization should further be evaluated.
